# Knowing that you know that you know? An extreme-confidence heuristic can lead to above-chance discrimination of metacognitive performance

**DOI:** 10.1093/nc/niae020

**Published:** 2024-05-22

**Authors:** Maxine T Sherman, Anil K Seth

**Affiliations:** Sussex Centre for Consciousness Science, University of Sussex, Brighton BN1 9QJ, United Kingdom; Department of Informatics, University of Sussex, Brighton BN1 9QJ, United Kingdom; Sussex Centre for Consciousness Science, University of Sussex, Brighton BN1 9QJ, United Kingdom; Department of Informatics, University of Sussex, Brighton BN1 9QJ, United Kingdom; Canadian Institute for Advanced Research, Program on Brain, Mind and Consciousness, Toronto M5G 1M1, Canada

**Keywords:** confidence, metacognition, meta-d

## Abstract

In daily life, we can not only estimate confidence in our inferences (‘I’m sure I failed that exam’), but can also estimate whether those feelings of confidence are good predictors of decision accuracy (‘I feel sure I failed, but my feeling is probably wrong; I probably passed’). In the lab, by using simple perceptual tasks and collecting trial-by-trial confidence ratings visual metacognition research has repeatedly shown that participants can successfully predict the accuracy of their perceptual choices. Can participants also successfully evaluate ‘confidence in confidence’ in these tasks? This is the question addressed in this study. Participants performed a simple, two-interval forced choice numerosity task framed as an exam. Confidence judgements were collected in the form of a ‘predicted exam grade’. Finally, we collected ‘meta-metacognitive’ reports in a two-interval forced-choice design: trials were presented in pairs, and participants had to select that in which they thought their confidence (predicted grade) best matched their accuracy (actual grade), effectively minimizing their quadratic scoring rule (QSR) score. Participants successfully selected trials on which their metacognition was better when metacognitive performance was quantified using area under the type 2 ROC (AUROC2) but not when using the ‘gold-standard’ measure m-ratio. However, further analyses suggested that participants selected trials on which AUROC2 is lower in part via an extreme-confidence heuristic, rather than through explicit evaluation of metacognitive inferences: when restricting analyses to trials on which participants gave the same confidence rating AUROC2 no longer differed as a function of selection, and likewise when we excluded trials on which extreme confidence ratings were given. Together, our results show that participants are able to make effective metacognitive discriminations on their visual confidence ratings, but that explicit ‘meta-metacognitive’ processes may not be required.

## Introduction

Perceptual metacognition, the process of reflecting upon perceptual decisions, is typically probed by testing whether individuals are able to accurately classify their judgements as correct or incorrect by obtaining subjective confidence ratings. When subjective decision confidence discriminates correct from incorrect choices, people are said to ‘know that they know’, i.e. to exhibit metacognitive sensitivity (Grimaldi et al. 2015). That this will occur is not trivial: under sufficient levels of sensory ambiguity confidence and accuracy can dissociate, such that the participant has no confidence in their above-chance performance (Peirce and Jastrow 1884).

The ability to form appropriate confidence judgements enables us to learn from our mistakes (Berg et al., 2016, Yeung and Summerfield 2012, Boldt and Yeung 2015) and update our beliefs (Stern et al. 2010), and it facilitates social communication and group decision-making (Zarnoth and Sniezek 1997, Frith 2012, Shea et al. 2014) by allowing us to communicate our beliefs and knowledge with degrees of (un)certainty. However, to navigate daily life, the mere estimation of subjective confidence is insufficient; we also need to know when confidence is predictive of future outcomes. For example, suppose a student feels sure they passed an exam with flying colours. If they know that they are poor at judging their exam performance, i.e. that their metacognition for exams is poor, then they would be ill-advised to adapt their behaviour according to that expected failure (e.g. by studying less for the next test). In order to know when to use confidence to guide future decisions, we need to be able to determine its reliability: we need to effectively judge our own metacognitive performance—to ‘know when we know when we know’.

Intuitively, it seems clear that we can evaluate the appropriateness of our confidence ratings for at least some tasks we perform in daily life, and this intuition was supported in a recently published study that used a simple visual decision-making task (Recht et al. 2022). Here, in a related study, we ask whether standard tasks and measures in the visual metacognition literature capture the ability to judge metacognitive performance. Our pre-registered study (https://osf.io/8vnuj) tested whether participants are able to distinguish between trials on which metacognition is good versus poor, and therefore whether people ‘know when they know when they know’. To do this, participants performed pairs of trials of a simple magnitude discrimination task, where each member of the trial pair was framed as an ‘exam’. On each trial they reported their confidence, in the form of a predicted exam grade (A–D). Once both trials had been completed, they had to select the trial on which they felt that their metacognition (the correspondence between their confidence and accuracy) was better. If metacognition is indeed higher on those selected trials than on unselected ones, then we can conclude that participants can discriminate their metacognitive ability on simple tasks used in the perceptual metacognition literature. In turn, this indicates that standard tasks and measures in the field pass a minimal test for ecological validity.

## Methods

### Participants

Participants were 39 naïve adults (33 female, 1 non-binary, 21 ± 6 years old) recruited from the University of Sussex Psychology database and reimbursed in course credits for their time.

We pre-registered the exclusion criterion that participants with accuracy outside the bounds of 60%–80% correct would be excluded, on the assumption that our staircase would achieve a mean accuracy of 70% correct. However, mean accuracy on our task was ∼75%, and this led to the removal of over 50% of our participants. We therefore changed our inclusion bounds to ±1 SD from the mean (62%–87.6%) and removed 5/4 participants for low/high performance, respectively.

We also pre-registered that participants would be excluded for insufficient variability in confidence reports (SD <0.2), in trial selection [P(select first trial) < 0.1 or > 0.9], or for excessive differences in accuracy between selected and unselected trials (accuracy for unselected <52% together with overall accuracy >60% suggesting purposeful failure on one of each trial pairs). No participants were excluded under any of those criteria. This left us with 30 participants for analysis.

All participants gave informed, written consent. The study received ethical approval from the University of Sussex ethics committee (C-REC).

### Stimuli and task

The task was presented on PsychToolbox (Brainard 1997, Kleiner et al. 2007) on a Linux computer running MATLAB 2017b, using a 17” LCD monitor (resolution) with 85 Hz refresh rate. Participants were sat approximately 60 cm from the screen.

**Figure 1.**
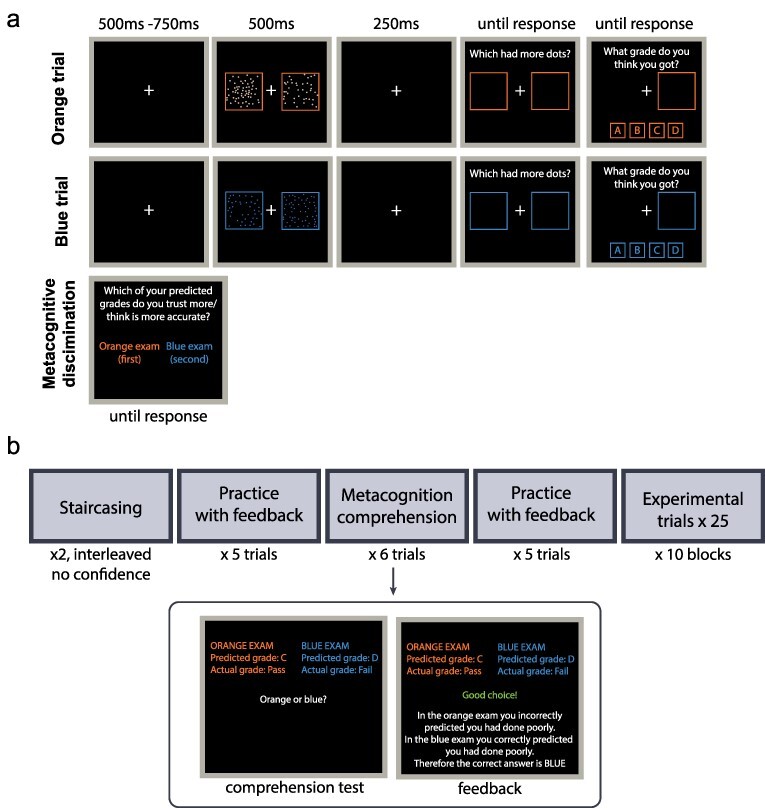
Experiment design.

The main task was a two-alternative forced-choice (2AFC) numerosity task (Fig. 1a). Trials began with the presentation of a white central fixation cross over a black background for 500–750 ms. Participants were presented with a random arrangement of dots (all in either orange or blue, see below) on the left and right of the screen. Dot arrays were presented in bounding boxes (7° × 9°), centred 5.5° away from fixation. On one side (randomly determined trial-by-trial), the number of dots was sampled from a normal distribution with a mean of 50 (SD = 5, truncated to have a min/max of 40/60 dots). On the other side, this number of dots was scaled by a ratio determined on an individual-subject basis to achieve ∼75% accuracy (for retained participants, ratio M ± SD = 1.20 + 0.06, or a difference in 9 ± 3 dots, see ‘Staircases’).

After 500 ms of presentation the dots were removed, and after a further 250 ms participants were taken to a response screen. The task was 2AFC numerosity discrimination: participants made (un-speeded) reports on whether there were more dots on the left or on the right side, by clicking inside the corresponding bounding box. Each trial was framed as an ‘exam’.

The selected bounding box (left/right) was highlighted (either orange or blue, see below and Fig. 1a), and then participants ‘predicted their exam grade’ as either A (highest grade), B, C, or D (fail). This prediction was provided by an un-speeded response in which participants clicked on the grade they thought they achieved. These predicted grades correspond to subjective confidence reports: a high (low) grade corresponds to the belief that the participant thought they performed well (poorly) on the numerosity task.

Numerosity trials were presented in pairs: first an orange trial was performed (all dots, bounding boxes, and text were presented in orange), and then a blue trial was performed (all dots, bounding boxes, and text were presented in blue). The side with more dots was randomly determined on each trial (and could vary across orange and blue trials), as was the positioning of the dots within the bounding box. Within a trial pair, the number of dots in each stimulus, N and N + δ, was always the same (i.e. difficulty was identical). While the values N and δ took varied pair-by-pair because of random sampling, difficulty was kept the same across trial pairs by keeping the ratio N:N + δ constant.

Following each trial pair, participants were asked to select the ‘exam’ in which they thought their predicted grade best matched their true grade: orange or blue. Notably, this decision is in principle orthogonal to both numerosity performance and predicted grade (confidence). For example, a participant might feel that her predicted grade of D (fail) matched performance on one trial (e.g. orange) better than her predicted grade of A matched performance on the other trial (blue) within a pair of trials. In other words, this response probes whether participants can judge their own metacognitive performance. In practice, this was two-interval forced-choice (2IFC) metacognitive discrimination where participants discriminated their metacognitive sensitivity over the previous two trials, where metacognitive sensitivity is defined according to the quadratic scoring rule (QSR):


$$QSR = 1 - {\ }{\left( {confidence - accuracy} \right)^2}.$$

### Staircases

The difference in dots between the left and right bounding boxes was determined by two interleaved 2 down 1 up psychophysical staircases. The starting difference was 150% and initial step size was 30%, reducing to 5% after two reversals. The staircases terminated after eight reversals. The dot difference was taken as the mean of the last four reversals of the two staircases.

Staircase trials were identical to the 2AFC numerosity task (‘exam’) described above, except participants neither predicted their grade nor made any metacognitive discriminations.

### Procedure

After giving informed consent, participants completed a staircase procedure that titrated the difference between the dots on the left and right of the screen. This took approximately 3 min.

Next, participants were given on-screen instructions for the full 2IFC experimental task, and completed five practice trials to familiarize them with the task. The task was framed as an exam. Confidence ratings were framed as ‘predicted grades’. The 2IFC metacognitive discrimination was explained as follows:

After you perform those two you will be asked to report the exam on which your predicted and true grade best match. In other words, which predicted grade do you trust the most? If for one of the exams you ‘know you did well’ OR you ‘know you DIDN’T do well’, pick that one.

After giving the 2IFC metacognitive report, participants were given feedback on their decision accuracy for both the orange and blue trials, and for their 2IFC report.

Next, participants completed six ‘comprehension’ trials (see Fig. 1b). Here, participants were presented with hypothetical accuracy and confidence reports for each exam and asked to select the exam on which metacognition was better. After making the choice, participants were given feedback and an explanation of the answer.

Next, participants completed seven more full practice trials with feedback, and then continued to the main experiment. No feedback was given on experimental trials. Participants completed 10 blocks of 25 trial pairs, and were offered a break after each block.

At the end of the 10 blocks, participants were fully debriefed and reimbursed for their time. The full testing session took approximately 50 min to complete.

### Measures of metacognition (pre-registered)

We pre-registered two measures of metacognition for this study: m-ratio and area under the type 2 ROC (AUROC2). M-ratio (Maniscalco and Lau 2012, Barrett et al. 2013, Fleming 2017) is a signal detection theoretic (SDT) measure (Green and Swets 1966) of metacognitive efficiency. Meta-d’ is the d’ (performance/sensitivity) required of an SDT-optimal observer who uses all available information to generate the participant’s pattern of confidence reports. A value of meta-d’ that exceeds d’ (i.e. m-ratio = meta-d’/d’ < 1) indicates that the optimal observer could achieve the same pattern of confidence responses with less Type 1 sensitivity, and therefore that the participant’s metacognition is suboptimal. Optimal metacognitive efficiency is indicated by meta-d’ = d’ (m-ratio = 1). Here, we estimated m-ratio using the MLE method of Maniscalco & Lau (Maniscalco and Lau 2012). Our pre-registration failed to mention how we would correct m-ratio estimates for empty cells. Consistent with our lab’s general approach (Sherman et al. 2015, Sherman et al. 2016a, 2016b, Sherman and Seth 2021), at the analysis stage, we used the rule by which 0.5 is added to all cell counts (hits, misses, false alarms, and correct rejections).

We also used a model-free measure: the area under the type 2 ROC (AUROC2) (Clarke et al. 1959, Galvin et al. 2003), which is a measure of metacognitive sensitivity (not efficiency). Here, analogously to Type 1 ROC curves, the Type 2 ROC consists of plotting P(confident| incorrect) against P(confident| correct) for each partition of the confidence scale, plus the points (0,0) and (1,1). The area under this curve gives AUROC2. When AUROC2 = 0.5, metacognitive performance is said to be at chance.

### Measures of metacognition (exploratory)

For completeness, alongside AUROC2, we considered the Kruskall-Gamma coefficient, G (Goodman and Kruskal 1954), and the phi correlation coefficient, Phi. Phi is identical to Pearson’s r when the data are binary. When data are not binary, Phi = χ^2^/n, where χ^2^ is the chi-squared statistic for the chi-squared test of independence and n is the number of participants. G is a non-parametric measure of association which reduces to a rank correlation in the binary case. Like AUROC2, both of these measures are confounded by Type 1 performance.

Next, we considered some recent extensions to AUROC2, Phi, and G: AUROC2-ratio, Phi- ratio, and G-ratio (Rahnev 2023). Here, and analogously to the measure m-ratio, their values are normalized by the value expected from the SDT-optimal observer that has the same sensitivity and decision threshold. Like m-ratio, these measures exhibit little dependence on Type 1 performance or response bias, though Phi- ratio is affected by metacognitive bias (Rahnev 2023).

Following Rahnev (2023), we took two further metacognitive measures derived from recent process models. The first, meta-uncertainty, quantifies second-order uncertainty in perceptual representations (Boundy-Singer et al. 2023). More specifically, Boundy-Singer and colleagues propose that confidence is based on the strength of the decision evidence—the distance between the decision variable and the decision threshold, normalized by the estimated standard deviation of sensory noise. This standard deviation is itself a random variable, whose standard deviation is called meta-uncertainty (uncertainty in the estimated perceptual uncertainty).

The second process model-based measure, meta-noise (Shekhar and Rahnev 2021), extends the standard Signal Detection Theory model into the metacognitive domain by assuming that the confidence criteria that straddle the decision threshold are subject to lognormal noise. Metacognitive (in)efficiency is then given by the variance of the fitted lognormal noise distribution.

All the above measures were estimated using code from Rahnev (2023) (https://osf.io/y5w2d/).

Finally, to quantify the accuracy of participant’s confidence ratings trial-by-trial, we used the Brier score (Brier 1950). In the metacognition literature, this is used for post-decision wagering and is called the QSR (Staël von Holstein 1970). We will refer to this measure as QSR, where QSR = 1–(confidence–accuracy)^2^. This quantity is effectively what participants were instructed to maximize during the experiment, and we use the difference in QSR between selected and unselected trials to evaluate participants’ ability to perform the task as instructed.

The reason for using it in this way, rather than as a measure of ‘meta-metacognition’ *per se*, is as follows: while above-chance identification of the higher-QSR interval might recruit metacognitive processes, it could also be achieved non-metacognitively. Because perceptual task performance is above chance, all else being equal, QSR increases with increasing confidence. Therefore, selecting the higher-confidence interval is a viable (and non-metacognitive) strategy for discriminating high from low QSR trials. This means that QSR cannot discriminate choosing the higher-metacognition trial from choosing the higher-confidence trial. We are not claiming that participants would use this strategy deliberately, nor that QSR cannot be used as a measure of metacognition within a trial. We only claim that it is a poor measure of ‘meta-metacognition’ in this paradigm.

### Statistics and stopping rule

Analyses were conducted on MATLAB using custom code (osf.io/8vnuj/files) and code provided in Rahnev (2023) (https://osf.io/y5w2d/).

After each trial pair, participants had to select the one in which their metacognition was higher. We grouped participant reports according to whether they came from ‘selected’ or ‘unselected’ trials. This grouping corresponds to ‘reported high metacognition’ and ‘reported low metacognition’, respectively. Then, both frequentist and Bayesian paired t-tests compared numerosity accuracy (% correct), mean confidence (estimated grade on a 4-point 0–1 scale), and metacognition (AUC2 and m-ratio) as a function of whether the trial was ‘selected’ or ‘unselected’. In exploratory analyses, we similarly compared the -ratio, meta-uncertainty, and meta-noise quantities across selected and unselected trials.

Priors for the Bayesian t-tests were uniform distributions from 0 to half of the theoretical maximum difference. This corresponds to a uniform distribution from 0 to 0.5 for confidence and m-ratio, which are theoretically bounded by 0 (chance) and 1 (maximum). Note that m-ratio can exceed 1 in practice (and may well do for ‘meta-metacognitive’ judgements), but the upper bound for m-ratio does not preclude finding evidence in favour for H1 if m-ratio values exceed 1. We used a uniform distribution from 0 to 0.25 for accuracy and AUC2, which are theoretically bounded by 0.5 (chance) and 1 (maximum).

Sample size was determined according to a Bayesian stopping rule. A minimum of 30 retained participants was required, and data collection continued until the Bayes factor (BF) for the difference in m-ratio between selected and unselected trials was sensitive (>3 or <1/3), or until we had 70 retained participants.

## Results

Participants’ ‘meta-metacognitive’ task in this experiment was to select the trial from each pair (orange or blue) in which they estimated their metacognitive performance to be higher.

Numerosity performance d’ did not significantly differ between the orange and blue trials [M_diff_ ± SE_diff_ = 0.01 ± 0.05, *t*(29) = 0.10, *P* = 0.922, d = 0.02]; however, participants did have a significant bias towards selecting blue trials (the second in the pair), making that choice 61.34 ± 1.42% of the time [t-test against 50%: *t*(29) = 8.01, *P* = < 0.001, d = 1.49].

For analysis, we categorized participant responses from each pair as ‘selected’ or ‘unselected’, corresponding to trials where participants rated their metacognitive performance as higher/lower, respectively.

**Figure 2.**
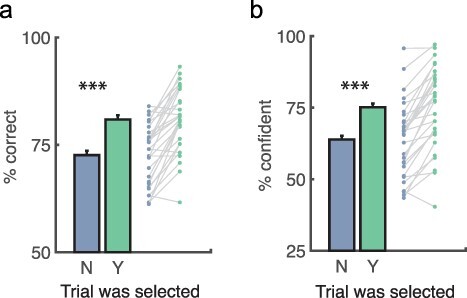
Task performance.

We expected that participants would select trials in which they performed better (higher accuracy), and this was confirmed (Fig. 2a): accuracy was on average 8% higher on selected than unselected trials, M_diff_ ± SE_diff_ = 8.29% ± 1.02%, t(29) = 8.16, *P* < 0.001, d = 1.52, BF_U[0,25]_ = 10^13^, RR = [0.24, inf]. We further expected that participants would be more likely to select trials on which their confidence was higher. Again, this was confirmed (Fig. 2c, d): confidence was approximately 11% higher on selected than unselected trials, M_diff_ ± SE_diff_ = 11.28% ± 1.35%, t(29) = 8.33, *P* < 0.001, d = 1.55, BF_U[0,50]_ = 10^14^, RR = [0.31, inf].

**Figure 3.**
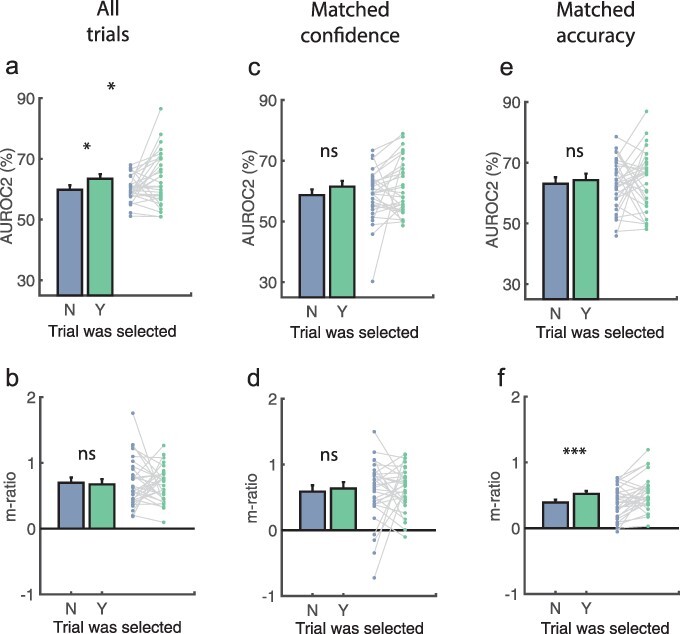
AUROC2 and m-ratio for selected and unselected trials.

So, on average, participants selected trials on which they were more confident. They also knew when they were correct, as indicated by their selecting trials on which their performance was higher. This is unsurprising. But was their metacognition higher on selected trials? Did they know when to trust their confidence ratings? Whereas there was anecdotal evidence for AUROC2 being higher on selected trials {Fig. 3a, M_diff_ ± SE_diff_ = 0.04 ± 0.02, t(29) = 2.37, *P* = 0.025, d = 0.44, BF_U[0,0.25]_ = 2.53, RR = [0.20, 1.94]}, m-ratio was equal {Fig. 3b, M_diff_ ± SE_diff_ = –0.02 ± 0.08, t(29) =–0.30, *P* = 0.763, d = -0.06, BF_U[0,0.5]_ = 0.27, RR = [0.39, inf]}. Results were therefore inconsistent across the model-free metacognitive sensitivity measure and the model-based metacognitive efficiency measure.

We expected that participants might adopt the strategy of betting on trials where their confidence was higher. To control for this, we ran the above analyses again using only trial pairs where participants gave the same confidence report (e.g. pairs where participants predicted a ‘B’ grade on both). Consistent with this concern, when participants could not use their confidence ratings as a cue, there was anecdotal evidence for AUROC2 no longer being higher for selected trials {M_diff_ ± SE_diff_ = 0.03 ± 0.02, t(29) = 1.45, *P* = 0.157, d = 0.27, BF_U[0,0.25]_ = 0.52, RR = [–inf, 0.39], see Fig. 3c}. Once again, m-ratio was largely equal {M_diff_ ± SE_diff_ = 0.05 ± 0.10, t(29) = 0.47, *P* = 0.639, d = 0.09, BF_U[0,0.5]_ = 0.38, RR = [–inf, 0.58], see Fig. 3d}.

In an exploratory control, we asked whether participants selected those trials on which they performed better, perhaps because those trials felt easier or were better attended to (which may be reasonable heuristics for estimating metacognitive sensitivity). As for the matched confidence analysis, when equalizing participants’ accuracy across trials AUROC2 no longer differed between selected and unselected trials {Fig. 3e, M_diff_ ± SE_diff_ = 0.01 ± 0.02, t(29) = 0.55, *P* = 0.587, d = 0.10, BF_U[0,0.25]_ = 0.18, RR = [0.14, inf]}. M-ratio was significantly *higher* for selected trials when equalizing accuracy {Fig. 3f, M_diff_ ± SE_diff_ = 0.13 ± 0.04, t(29) = 2.95, *P* = 0.006, d = 0.55, BF_U[0,0.50]_ = 17.19, RR = [0.03, 2.90]}. However, this m-ratio effect is likely an artefact of almost all responses in this subset being correct responses (90.2% ± 6.4%).

## Participants effectively select trials on which confidence is best aligned to accuracy (exploratory)

Our research question asked whether people are able to determine when their metacognition is good versus poor. The analysis so far has indicated that this was the case when using AUROC2 but not m-ratio as our measure, and that when confidence was controlled for, neither metacognitive measure was higher on selected trials. Was this because participants were not performing the task as we intended?

**Figure 4.**
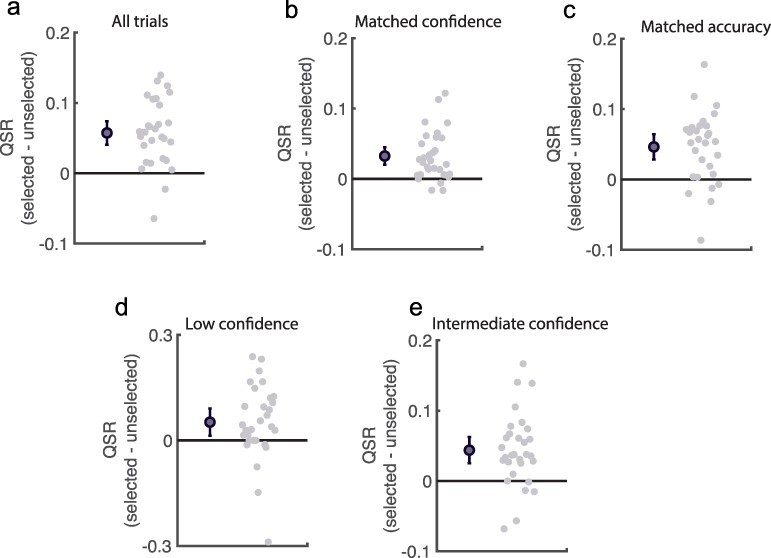
Trial-by-trial assessment of metacognitive performance.

To test this we examined participants’ trial-by-trial behaviour in more detail. Participants’ task was effectively to discriminate their QSR (broadly, the difference between their confidence and accuracy): were they able to do this? QSR was indeed higher on selected than unselected trials, M_diff_ ± SE_diff_ = 0.06 ± 0.01, t(29) = 6.71, *P* < 0.001, d = 1.25 (Fig. 4a), even when their confidence on both pair items was identical, M_diff_ ± SE_diff_ = 0.03 ± 0.01, t(29) = 5.11, *P* < 0.001, d = 0.95 (Fig. 4b) and when accuracy was identical, M_diff_ ± SE_diff_ = 0.05 ± 0.01, t(29) = 5.07, *P* < 0.001, d = 0.94 (Fig. 4c). Mirroring this pattern of higher QSR scores on selected trials, participants selected high QSR trials (i.e. made ‘good selections’) on 68.33% ± 2.03% of trials (t-test against 50%, *P *< 0.001, d = 1.68), again, even when confidence ratings were the same across pairs (59.84% ± 2.20%, *P* < 0.001, d = 0.83).

Therefore, participants were indeed able to identify those trials on which their metacognition - as measured by QSR scores - was higher, even when they were unable to use their confidence report as a cue.

For the majority of trial pairs (80.90%), numerosity responses on selected trials were correct. But were participants sensitive to cases where their confidence was appropriately ‘low’ when they made incorrect decisions? In other words, do participants ‘know that they know that they “don’t” know’? In this even stricter test, we restricted analyses to pairs on which participants selected the trial where their confidence was lower. Note that in most cases participants did the opposite (see Fig. 2b). If participants know when they know when they ‘don’t know’, then when participants selected the more uncertain trial response QSR should remain higher than on those unselected, higher confidence trials. This was indeed the case: metacognition as measured with QSR remained better on the (lower confidence) selected than (higher confidence) unselected trials, M_diff_ ± SE_diff_ = 0.05 ± 0.02, t(29) = 2.62, *P* = 0.014, d = 0.49 (Fig. 4d), meaning that participants could judge when their low confidence reports were reliable as well.

Taken together, these results suggest that participants performed the task as instructed, and were able to discriminate trials on which their QSR was better versus worse, despite this not being well-reflected in AUROC2 and m-ratio differences.

## No summary measure of metacognition is higher on selected trials when confidence is matched (exploratory)

**Figure 5.**
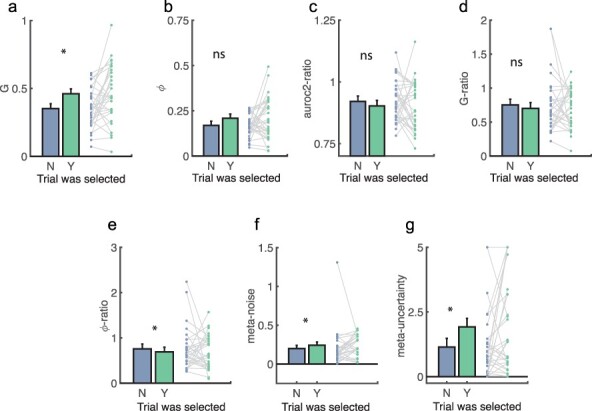
Selected vs unselected trials: other measures of metacognitive sensitivity and efficacy, calculated over all trial pairs.

Despite participants being able to perform the task as instructed, when reported confidence was matched we did not find compelling evidence that participants could discriminate the trials on which their metacognition (as measured by AUROC2 and m-ratio) was better. We explored this further by considering a wider range of metacognitive efficiency measures recently proposed and/or psychometrically described in Rahnev (2023): AUROC2-ratio, gamma-ratio, and phi-ratio, as well as model-based measures meta-uncertainty and meta-noise (see Methods for details). All of these measures are robust to changes in Type 1 performance (Rahnev 2023). Finally, we further considered two measures of metacognitive sensitivity to mirror our analyses on AUROC2: phi and gamma, neither of which is robust to changes in Type 1 performance. As before, each of these measures was computed separately for ‘selected’ and ‘unselected’ trials. We also calculated them on trials where confidence was matched (see Fig. 5 and Table 1).

**Table 1.** Alternative metacognitive measures (selected—unselected)

	All trials			Matched confidence		
	M_diff_ ± SE_diff_	W	*P*	M_diff_ ± SE_diff_	W	*P*
G	0.11 ± 0.04	355	**0.012**	0.09 ± 0.08	298	. 178
Phi	0.04 ± 0.02	296	0.192	0.03 ± 0.03	272	. 417
AUROC2-ratio	−0.02 ± 0.02	178	0.262	0.00 ± 0.03	218	. 766
G-ratio	−0.05 ± 0.09	221	. 813	0.10 ± 0.17	240	. 877
Phi-ratio	−0.07 ± 0.11	207	0.600	0.07 ± 0.17	235	. 959
Meta-noise	0.04 ± 0.04	319	**0.028**	0.04 ± 0.03	209.50	. 388
Meta-uncertainty	0.78 ± 0.34	331	**0.043**	−0.07 ± 0.31	204	. 558

Statistically significant p-values (at α < .05) are highlighted in bold font

As can be seen in Fig. 5 the measures were unstable for several participants (as indicated by the extreme values taken), so for each we compared selected and unselected trials by means of Wilcoxon sign rank tests.

As shown in Table 1, the only measures that discriminated selected and unselected trials were Phi (*P* = 0.012 uncorrected), meta-noise (*P* = 0.028 uncorrected), and meta-uncertainty (*P* = 0.043 uncorrected), and none of these results remained significant when restricting analyses to trial pairs with equal confidence ratings. That the novel -ratio measures were not different between selected and unselected trials is unsurprising given that they are measures of metacognitive efficiency like m-ratio.

## An extreme-confidence heuristic? (exploratory)

**Figure 6.**
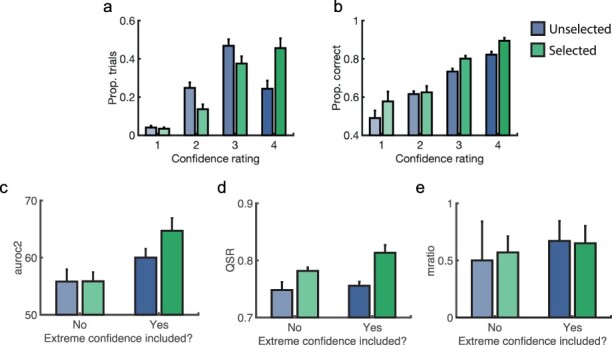
Testing for an extreme confidence heuristic.

If participants use confidence ratings to determine their selections, do participants simply select the trial on which their confidence was more extreme (1 or 4 vs 2 or 3)? This would be a sensible heuristic with which to complete the task because extreme confidence ratings are less uncertain. To address this, first, for each participant and separately for selected and unselected trials, we calculated the proportion of trials on which each confidence rating was reported. As can be seen from Fig. 6a, there was indeed an upwards shift in the distribution of reported confidence for selected versus unselected trials. This was not accompanied by a shift in accuracy (Fig. 6b).

Next we tested for the ‘extreme confidence heuristic’ more explicitly, taking the subset of trial pairs in which the two confidence reports were equally extreme (reported confidence was ‘2’ or ‘3’). In this subset, the heuristic cannot be applied. Therefore, if participants do select the extreme confidence trial as their ‘higher metacognition’ trial, then here metacognitive performance should be equal (or at least reduced) across selected and unselected trials. We recalculated AUROC2, m-ratio, and QSR for selected and unselected trials, using only those trial pairs where confidence ratings intermediate. Then, by means of 2 × 2 repeated-measures ANOVAs, we compared each measure as a function of whether the trial was selected or not, and whether each calculation included extreme ratings (‘1’ and ‘4’) or not (‘2’ or ‘3’ only). Note that we were unable to compare against trial pairs with only extreme confidence ratings because there were not enough such pairs in the dataset.

Four participants had fewer than 40 trials in this subset (*N* = 6, 9, 19, and 38) so they were excluded. Though this threshold of 40 trials is somewhat arbitrary, results were consistent under all more conservative thresholds (e.g. *N* = 150).

The difference in AUROC2 between selected and unselected trials was indeed reduced when excluding extreme ratings [interaction F(1,25) = 5.63, *P* = 0.026, Fig. 6c], and was reduced to zero {t-test without extreme confidence, *t*(25) = 0.03, *P* = 0.973, BF_H(0,0.05)_ = 0.32,^1^ RR = [0.05, -inf]}. This supports the hypothesis that meta-metacognitive performance on this task is facilitated by the ability to choose trials on which extreme confidence ratings were given. The difference in QSR was reduced too [interaction F(1,25) = 18.13, *P* < 0.001, Fig. 6d], though remained above 0 {t(25) = 3.82, *P* < 0.001, BF_H(0,0.06)_ = 189, RR = [0, 4.18]}.

The difference in m-ratio as a function of selection was not significant [interaction F(1,25) = 0.15, *P* = 0.699, Fig. 6e], which is unsurprising given that it was invariant to selection choice in the first place.

To summarize, these results suggest that at least to some degree, participants use an extreme-confidence heuristic to complete the task.

## Replication using data from Recht et al. (2022)

In a recent study, Recht et al. (2022) used a similar experimental design to test whether participants ‘know when they know when they know’: 12 expert participants performed 400 trial pairs (800 total) of a 2AFC spatial frequency discrimination with confidence (metacognition) and, after each, performed a 2IFC discrimination on their metacognition (meta-metacognition). Unlike here, participants further reported confidence in their 2IFC report (meta-meta-metacognition).

We used these data to address the question of whether our findings would replicate in experienced observers, in case the lack of an effect under m-ratio was driven by our failure to adequately explain to participants how to evaluate ‘meta-metacognition’. Accordingly, we repeated the analyses above on the Recht et al.’s dataset.

**Figure 7.**
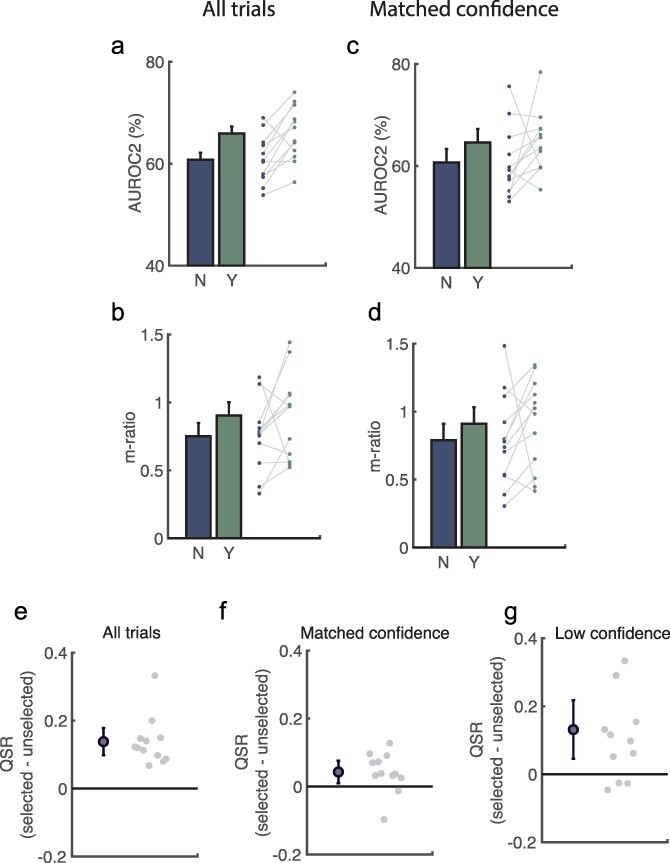
Reanalysis of the Recht et al. (2022) dataset.

All our findings were largely replicated, at least qualitatively (see Table 2 for all results). In particular, AUROC2 was higher in selected than unselected trials, *t*(11) = 3.67, *P* < 0.001 (Fig. 7a), but m-ratio was not, *t*(11) = 1.52, *P* = 0.157. When confidence was matched across selected and unselected trials, AUROC2 no longer differed, *t*(11) = 1.44, *P* = 0.177 (Fig. 7c). Results under m-ratio remained non-significant, *t*(11) = 0.99, *P* = 0.343 (Fig. 7d). QSR scores remained higher for selected than unselected trials, *t*(11) = 6.73, *P* < 0.001 (Fig. 7e), even when confidence was matched, *t*(11) = 5.15, *P* < 0.001 (Fig. 7f), and even when participants selected the trial on which confidence was lower, *t*(11) = 2.99, *P* = 0.012 (Fig. 7g). We interpret this as demonstrating that participants were able to make the discrimination as instructed in all control analyses (see Methods for justification), though we appreciate that this could also be interpreted as above-chance discrimination of metacognitive performance. When running analyses on the set of alternative metacognitive measures, none were significantly different when confidence was matched (all W ≤ 63, all *P* > 0.064). These results support the notion that our results are not caused by a failure to understand task instructions.

**Table 2.** Re-analysis of the Recht et al.’s dataset (selected—unselected)

	All trials			Matched confidence			Matched accuracy		
	M_diff_ ± SE_diff_	t	*P*	M_diff_ ± SE_diff_	t	*P*	M_diff_ ± SE_diff_	*t*	*P*
% correct	5.62 ± 1.88	2.99	0.001						
% confident	17.1 ± 2.46	7.00	**<0.001**						
Auroc2	0.05 ± 0.01	3.67	**<0.001**	0.04 ± 0.03	1.44	. 177	0.04 ± 0.02	2.11	0.059
m-ratio	0.15 ± 0.10	1.52	. 157	0.12 ± 0.12	0.99	. 343	0.05 ± 0.01	3.78	**0.003**
QSR	0.64 ± 0.02	6.73	**<0.001**	0.58 ± 0.02	5.15	**<0.001**	0.58 ± 0.02	8.14	**<0.001**
Alternative measures of metacognition									
	M_diff_ ± SE_diff_	W	*P*	M_diff_ ± SE_diff_	W	*P*			
G	0.16 ± 0.06	67	0.027	0.11 ± 0.08	58	0.151			
Phi	0.09 ± 0.03	68	0.021	0.07 ± 0.05	62	0.077			
Auroc2-ratio	0.05 ± 0.03	60	0.110	0.04 ± 0.04	63	0.064			
G-ratio	0.19 ± 0.09	65	0.042	0.16 ± 0.11	61	0.092			
Phi-ratio	0.19 ± 0.10	62	0.077	0.18 ± 0.14	63	0.064			
Meta-noise	−0.20 ± 0.24	24	0.266	−0.31 ±0.28	22	0.204			
Meta-uncertainty	−0.16 ± 0.46	29	0.470	−0.31 ±0.55	18	0.110			

Statistically significant p-values (at α < .05) are highlighted in bold font

## Discussion

Here, we tested whether human participants can discriminate trials in which their metacognition was better or worse. Results under AUROC2 (a condition-average, model-free measure of metacognitive sensitivity) supported the notion that individuals indeed were able to do so, in line with recent findings by Recht et al. (2022). The same was found under exploratory analyses on two other model-based measures: meta-noise (Shekhar and Rahnev 2021) and meta-uncertainty (Boundy-Singer et al. 2023). However, we did not find this result when comparing m-ratio, nor when running exploratory analyses on other metacognitive efficiency measures (Rahnev 2023).

At first glance, one might think the most compelling explanation for this discrepancy is that m-ratio (and other -ratio measures) is a poor measure to address this question because, by design, it is invariant to precisely those sources of information that participants would use to complete the task: it is (largely) invariant to changes in perceptual sensitivity, response bias, and confidence bias. M-ratio reflects, more or less, the degradation (or enhancement) of the evidence available for metacognitive evaluation, and accordingly is not a quantity which humans can necessarily ‘read-out’ or evaluate.

The problem with this explanation is that while participants’ AUROC2 was higher on those trials selected as ‘better metacognition’ trials, this was not the case when the same confidence rating was given in both instances of the trial pair. In exploratory analyses on alternative measures of metacognition, we again found that none of the three summary metacognitive sensitivity measures, four metacognitive efficiency measures, and two process model-based measures we tested distinguished selected from unselected trials when confidence ratings were matched. This suggests that the ability to evaluate the quality of metacognitive ratings is impaired when participants are unable to use their reported confidence as a cue. In other words, the ability to judge one’s own metacognitive performance (at least in this paradigm) can be accomplished, at least in part, heuristically, without superordinate ‘meta-metacognitive’ processes.

It is worth noting that the cue-based approach to the task was not driven by participants’ failure to understand or perform the task. Their trial selections (i.e. the ‘meta-metacognitive’ choices) were entirely in line with the task instructions: QSR, the quantity participants were effectively instructed to minimize, was higher for selected trials even when confidence was equal across the pair, and even when participants selected the trial on which confidence is lower. In other words, participants were able to report as though they ‘know when they know that they “don’t” know’, indicating that they could make effective judgements on both high and low confidence reports.

One way in which participants could accomplish this without ‘meta-metacognitive sensitivity’ would be by selecting the trial on which their confidence was most extreme (here, ‘1’ or ‘4’). The idea here would be that extreme confidence reports are low-uncertainty confidence reports. This would be a heuristic approach to achieving above-chance ‘meta-metacognition’ that does not require the ability to evaluate metacognitive inferences via introspection. In support of this heuristic strategy, we found that the difference in AUROC2 between selected and unselected trials was reduced in trial pairs where only intermediate (‘2’ or ‘3’) confidence ratings were given. In other words, when participants could not use this extreme confidence heuristic because the two confidence ratings were equally extreme, ‘Type 3’ performance was impaired. The difference in QSR between selected and unselected trials also decreased, though it remained above zero. Together, these results suggest a potential role of heuristics in guiding ‘meta-metacognitive’ discriminations.

These results are consistent with recent work by Zheng et al. (Zheng et al. 2023). There, participants made a binary Type 1 choice, then binary confidence (confident/guess), and finally were asked either ‘Are you absolutely not confident, i.e. no idea which answer is correct, or do you have at least an inkling about the correct answer?’ or ‘Are you absolutely confident, i.e. knowing the correct answer for certain, or do you just have a strong feeling that you are correct this time’, depending on their Type 2 response. The final response was considered a ‘Type 3’ rating (judgement on confidence), though arguably it could also be interpreted as an instruction to increase the granularity of the Type 2 report. These subjective ratings were compared to ratings made in a condition where participants simply gave their Type 1 response followed by confidence on a 4-point scale. Metacognition as measured by m-ratio was comparable across the two conditions, which the authors interpreted as showing that meta- and meta-metacognitive ratings arise from the same system. The idea that ‘Type 3’ ratings are generated by ‘Type 2 processes’ is entirely consistent with what we propose here.

Our paradigm is very similar to that used in a recent paper by Recht et al. (2022) (though designed independently, see pre-registration at https://osf.io/8vnuj). There, and using experienced participants, the authors also had participants perform a 2AFC perceptual task with confidence ratings and then a 2IFC task to select the interval with higher metacognition. That 2IFC report, unlike here, had a further confidence judgement associated with it (a ‘Type 4 report’ or meta-meta-meta-cognition). The authors found that even ‘Type 4’ sensitivity was above chance. Our results are consistent with the authors’ findings in supporting the idea that participants can successfully report on the quality of their confidence ratings in simple visual tasks. However, repeating our analysis pipeline on their dataset revealed convergent results. Most importantly, while AUROC2 was higher for selected than unselected trials across their dataset, this was no longer the case when confidence was matched (an analysis not performed by Recht and colleagues). As was the case in our data, QSR remained significantly higher in selected trials, but a difference in QSR is not compelling evidence for above-chance ‘meta-metacognitive’ performance because it is possible to achieve using heuristics alone (please see Methods for details).

Taken together, we have shown, consistent with other recent work (Recht et al. 2022, Zheng et al. 2023), that human participants performing a simple visual task are able to discriminate the quality of their confidence reports, i.e. report when their confidence is a better versus worse predictor of their accuracy. However, participants may be able to make these discriminations, at least in part, through a heuristic approach which compares how extreme confidence ratings. It is worth emphasizing that accomplishing the task heuristically does not mean that participants cannot do the task, nor that they do so inefficiently. On the contrary, heuristics can be efficient and adaptive solutions to solving complex or effortful tasks (Gigerenzer and Gaissmaier 2011). For example, the notion of retrieval fluency is commonly recruited to explain how we make metacognitive judgements about our memory knowledge (Schwartz et al. 1997, Benjamin et al. 1998). As the field of visual metacognition expands outside of simple perceptual tasks to more ecologically valid tasks of higher complexity (Rahnev et al. 2022), it may be that heuristic accounts of metacognitive judgements are increasingly favoured over models centred on statistical or probabilistic computations.

## Data Availability

All data and analysis code are freely available to download at https://osf.io/8vnuj.

## Footnotes

1 The prior for the Bayes Factors are taken as the mean difference over all trials.
